# Determinants and Patterns of Contraceptive Use among Sexually Active Women Living with HIV in Ibadan, Nigeria

**DOI:** 10.21203/rs.3.rs-3993771/v1

**Published:** 2024-03-06

**Authors:** Folahanmi Tomiwa. Akinsolu, Zainab Oyindamola Adegbite, Samuel A. Bankole, Abisola Lawale, Ifeoluwa Eunice Adewole, Olunike Rebecca Abodunrin, Mobolaji Timothy Olagunju, Oluwabukola Mary Ola, Abel Nnamdi Chukwuemeka, Aisha Oluwaseun Gambari, Jacinta N. Nwogu-Attah, Hiliary Nosa Okunbor, Akim Tafadzwa Lukwa, Ebiere Herbertson, George Uchenna Eleje, Olatunji Akanni, Oliver Chukwujekwu Ezechi

**Affiliations:** Lead City University; Lead City University; Lead City University; Lead City University; Lead City University; Lead City University; Nanjing Medical University; Lead City University; Lead City University; Lead City University; Lead City University; Babcock University Teaching Hospital; University of Cape Town; Nigerian Institute of Medical Research; Nnamdi Azikiwe University Teaching Hospital; Lead City University; Lead City University

**Keywords:** Contraceptive, Sexually Active Women, Human Immunodeficiency Virus

## Abstract

**Background:**

Contraception is a strategy to meet the family planning goals of women living with human immunodeficiency virus (WLHIV) as well as to reduce the transmission of HIV. There is limited data from Nigeria, where HIV prevalent is the second-largest in the world. This study aimed to examine contraceptive use and identify factors influencing its use among sexually active WLHIV in Ibadan, Nigeria.

**Methods:**

A facility-based cross-sectional study was conducted among 443 sexually active WLHIV across three HIV treatment centers in Ibadan, Oyo State. The inclusion criteria were WLHIV, aged 18–49 years, who asserted being fecund and sexually active. An adopted questionnaire was used to collect data, and the data was analyzedusing the Statistical Package for Social Sciences (SPSS) Windows version 25. Statistical significance was set at p < 0.05.

**Results:**

Among sexually active WLHIV (n = 443), 73.1% used contraceptives, with 26.9% having unmet needs. The results revealed a significant association between employment status and the use of contraceptives (AOR = 2.150; 95% CI 1.279–3.612 p=0.004); accessibility to contraceptive methods and the use of contraceptives (AOR = 21.483; 95% CI 7.279–63.402 p=0.00). Also, a significant association was found between payment for service and contraceptive use (AOR = 14.343; 95% CI 2.705–76.051; p = 0.003). Previous reactions towards contraceptive use were also significantly associated with contraceptive use (AOR = 14.343; 95% CI 2.705–76.051 p = 0.003). The dual contraceptives usage rate was 30.7%.

**Conclusions:**

Although contraceptive use among sexually active WLHIV was high, the study highlighted the need for increased adoption of dual contraceptive methods to mitigate the risk of unintended pregnancy and HIV re-infection among this population. It emphasized the importance of continuous sensitization and counseling services healthcare providers provide to promote contraceptive use among WLHIV.

## INTRODUCTION

Globally, there are about 37.7 million Human Immunodeficiency Virus (HIV)-positive individuals worldwide, of which 20.2 million are women living with HIV. [1, 2] It has been determined that Sub-Saharan Africa is the area most severely affected by the HIV epidemic, accounting for more than two-thirds of all HIV infections worldwide. Nigeria has the second-largest HIV epidemic in the world and the region’s highest incidence of new infections. In Nigeria, 1.7 million people lived with HIV (PLHIV) in 2020. [3] Women can make independent choices about having children and having sex, regardless of their HIV status. Women living with HIV (WLHIV) must be informed, given the freedom to choose a safe, effective method of contraception, and provided with it at nearby health centers.[4] Because it prevents unplanned pregnancies, contraception may also be essential for preventing Mother-to-Child Transmission of HIV. [5] Strengthening contraceptive programs is therefore essential to lower the high incidence of unintended pregnancies, which could contribute to the elimination of HIV/AIDS outbreaks by 2030 (Sustainable Development Goal 3.3). [6, 7]

There appears to be a substantial unmet need for contraception among WLHIV and contraceptive failure due to the high incidence of unwanted pregnancy and abortion.[8] Nigeria accounts for 70% of sexually active WLHIV, 5.5 million WLHIV births annually, and 15% of the world’s low contraceptive uptake.[9] Furthermore, prior research found that Nigerian WLHIV had a high level of awareness about contraception, but that knowledge did not match their use of contraceptives, which was linked to a high proportion of unwanted pregnancies.[10] Women who are fecund, sexually active, and who report not wanting any more children or wishing to delay the next child are considered to have unmet needs, according to the WHO.[11] Because ART has increased the overall survival rate of PLHIV, WLHIV must have defined reproductive life plans that include accessible access to contraception. Most of the research that has been done on contraceptive use among WLHIV has focused on married women. [12] Because of this, sexual activity among sexually active unmarried women puts them at a higher risk of having an unexpected child because of the idea that sex can only take place within the setting of a married relationship. Therefore, there is a lack of reproductive plans for these women through health services. [13–15]

Although the WHO has recommended that WLHIV have the right to choose any method of contraception, similar to HIV-negative women, the choice of contraception in the presence of HIV appears to be more complicated because WLHIV is required to strike a balance between the prevention of unintended pregnancy and HIV transmission. [16, 17] Sexually active WLHIV can also plan their reproductive lives to avoid unwanted pregnancies and enjoy parenthood like their counterparts who are not living with HIV. Hence, this study aims to identify key predictors of contraceptive use among sexually active WLHIV in Ibadan, Oyo State, Nigeria.

## MATERIALS AND METHODS

### Study Design and Setting

#### Design Overview

The research employed a facility-based cross-sectional survey approach. In a cross-sectional survey, researchers observe and collect data from participants simultaneously rather than over a prolonged period. This study design is beneficial for understanding the prevalence of a phenomenon, condition, or opinion at a given moment. [18]

#### Target Population

The study specifically focused on sexually active women living with WLHIV. By targeting this particular group, the research aimed to gain insights relevant to their experiences, challenges, and needs.

#### Geographical Setting

The study was conducted in Ibadan, Oyo State, Nigeria. Oyo State, located in the southwestern part of Nigeria, is one of the country’s significant states, with a rich cultural heritage and diverse population. Understanding the experiences of WLHIV in this region could provide insights that might reflect broader trends within the state or even the country, given its diverse demographic makeup.

#### Facility Details

Three prominent HIV treatment centers in Oyo State were chosen as the study sites:

1. *Adeoyo Maternity Hospital Yemetu*: Located in the Yemetu area, this maternity hospital is a haven for expectant mothers and a pivotal center providing HIV treatments and counseling services. It’s a reputable institution known for its comprehensive care for women, especially those living with HIV.
2. *State Hospital Adeoyo Ring Road*: Situated on the bustling Ring Road, this state hospital is one of the primary healthcare providers in the region. It caters to a wide range of medical needs, with a particular unit dedicated to HIV treatments, ensuring patients receive both medical and emotional support.
3. *St. Annes Anglican Hospital Molete*: A faith-based institution in Molete, St. Annes Anglican Hospital balances spiritual and medical care. Their commitment to serving those living with HIV makes them a crucial part of the healthcare landscape in Oyo State.

#### Study Timeline

This study’s data collection and observations were carried out over three months, from September to November 2022. This timeframe was chosen to ensure comprehensive data collection while minimizing seasonal or temporal biases that might affect the participants’ responses.

### Study population and sampling

#### Purposive Sampling for Facilities

A purposive sampling technique was utilized for this study. Purposive sampling is a non-probabilistic method where researchers select particular groups or individuals for their specific qualities or characteristics. [19] In this case, the health facilities were chosen based on antiretroviral therapy (ART) treatment availability. Not all health facilities offer ART treatment; hence, selecting those that specifically provide this service was vital to ensure the study’s participants were relevant to the research objectives.

#### Random Selection of Participants

Once the facilities were determined, the actual participants for the study were chosen through a random sampling process within each facility. This method ensures that each eligible participant in the selected health facilities has an equal chance of being chosen for the study, minimizing biases and ensuring the results represent the target population within those facilities. [20]

#### Inclusion Criteria

Participants were included in the study based on the following set of criteria:

1. They identified as WLHIV.
2. They were aged between 18 and 49 years, ensuring the study focused on the reproductive age group.
3. They had been sexually active within the last six months.

This criterion was crucial to understanding the current sexual behaviors and practices among WLHIV.

#### Study Flowchart and Numbers

A comprehensive flowchart was designed to map out the participant selection process. Initially, 750 WLHIV from the selected facilities were approached and interviewed. However, after applying the inclusion criteria, particularly the requirement of recent sexual activity, the final sample size was narrowed down. Out of the initial 750 participants, 443 sexually active WLHIV were found to meet all the study’s criteria and were subsequently analyzed in-depth.

### Data Collection

Data was collected using adapted questionnaires from a previous study on contraceptive use among sexually active WLHIV. [17] The questionnaire was divided into three sections: Socio-Demography Data Questionnaire among sexually active WLHIV, Contraceptive Use Questionnaire among sexually active WLHIV, and Perceived factors influencing Contraceptive Use Questionnaire among sexually active WLHIV. Before the main study, the adapted questionnaire was pretested on a representative sample, feedback on clarity and relevance was collected, and statistical analyses, including Cronbach’s Alpha, affirmed their internal solid consistency (α = 0.85) and validity, ensuring their suitability for the research objectives. [21] The selected participants were those who voluntarily consented to participate. All research assistants were trained before the commencement of the study on the research tools, interviewing skills, data management, and clarifications of ethical issues in research. The research assistants administered the questionnaires in English or the local language to participants who could neither read nor write. The questionnaires were administered privately, and clarification and assistance were provided where necessary. The interviews took approximately 20 minutes to complete.

### Statistical Analysis

The data gathered from the comprehensive questionnaires was diligently processed and meticulously analyzed utilizing the *Statistical Package for Social Science (SPSS) version 25*. SPSS is a widely recognized and powerful software for handling and analyzing statistical data, particularly in social sciences research. Variables in the dataset were assessed to determine if they were normally distributed or not to determine the appropriate statistical analysis to be used. Mean was used to describe the customarily distributed variables, while the median was used for the non-normally distributed variables. Linear regression was used to assess the statistical association between variables that had categorized outcomes more than 3.

Meanwhile, logistic regression assessed the statistical association between selected variables and variables with dichotomized outputs such as contraceptive use: Yes/No, True/False. The adjusted regression models included covariates such as age, education level, marital status, income level, and others. The strength of the association was assessed by setting a significance level at p-value < 0.05.

### Ethical Considerations

The Health Department of Planning Research & Statistics Division, Oyo State Ministry of Health, provided the Ethics Approval for this study (AD 13/479/44542A). Official permission was obtained from hospitals included in this study. The verbal/written consent procedure was conducted in a separate and private room, administered by trained data collectors. The study participants were assured that their involvement was entirely voluntary, and they retained the right to decline participation or revoke their consent at any juncture.

Significantly, it was stressed that their participation would not impact the medical care they received. Participants were also apprised that the survey might entail sensitive or personal inquiries related to reproductive health concerns, which could be uncomfortable or distressing. Also, participants were explicitly informed that they were under no obligation to answer any question that they found uncomfortable and had the liberty to withdraw from the study or choose not to respond to specific questions at any point. In cases where participants required emotional support, female nurses were available to offer psychological assistance. All collected data were transformed into an anonymized format and stored on laptops protected by passwords throughout the data collection. Furthermore, the data was stored on secure, password-protected computers to ensure confidentiality and security.

## RESULTS

### Socio-demographic Characteristics of Participants

Table 1 provides a detailed overview of the socio-demographic characteristics of the study participants of 433 individuals. Among the participants, 324 (73.1%) were contraceptive users, while 119 (26.9%) were non-users of contraceptives. Regarding age, the average age of contraceptive users was 36.62 years, with a standard deviation of 6.70 years. In contrast, non-users had a slightly higher average age of 37.59 years with a standard deviation of 7.20 years. When examining marital status, a majority of contraceptive users were married, accounting for 74.6% (290 individuals). The proportions of divorced, widowed, separated, and single users were 70.0%, 56.2%, 66.7%, and 62.5%, respectively. On the other hand, among non-users, 99 (25.4%) were married, 3 (30.0%) were divorced, 7 (43.8%) were widowed, 4 (33.3%) were separated, and 6 (37.5%) were single.

Ethnic distribution showed that the Yoruba ethnic group constituted the majority of contraceptive users at 75.0% (285 individuals). Other ethnic groups among users were Igbo at 62.5%, Hausa at 50.0%, and others at 72.7%. For non-users, the Yoruba, Igbo, Hausa, and other ethnic groups constituted 25.0%, 37.5%, 50.0%, and 27.3%, respectively. In terms of religion, 188 (72.0%) of the contraceptive users identified as Christians, and 136 (74.7%) identified as Muslims. Among the non-users, 73 (28.0%) were Christians, and 46 (25.3%) were Muslims. For educational level, among contraceptive users, 93 (78.8%) had primary education, 135 (67.8%) had secondary education, 65 (78.3%) had tertiary education, and 31 (72.1%) had no formal education. Among non-users, the percentages for primary, secondary, tertiary, and none were 21.2%, 32.2%, 21.7%, and 27.9%, respectively.

Regarding employment status, a substantial proportion of contraceptive users, 273 (76.5%), were employed, whereas 51 (59.3%) were unemployed. In contrast, 84 (23.5%) of non-users were employed, and 35 (40.7%) were unemployed. About the type of partner, 269 (74.9%) of contraceptive users had a spouse, 32 (80.0%) had a steady partner, 14 (53.8%) had a casual partner, and 9 (50.0%) had no partner. For non-users, the distribution was 90 (25.1%) with a spouse, 8 (20.0%) with a steady partner, 12 (46.2%) with a casual partner, and 9 (50.0%) with no partner. Considering the monthly income of the participants, 77.6% of contraceptive users (N=225) reported earning less than 44.71 USD (NGN 33,000), while 22.4% of non-users (N=65) fell within this income bracket. On the other hand, 64.7% of contraceptive users (N=99) earned 44.71 USD (NGN 33,000) or more, compared to 35.3% of non-users (N=54) with a similar income level. (See Table 1)

### Level of Dual Contraceptive Utilization

Table 2 presents data on the contraceptive utilization patterns among the participants. Out of the participants, 30.7% (N=136) reported using dual contraceptives. Meanwhile, 42.4% (N=188) indicated that they use a single contraceptive method. Notably, 26.9% (N=119) of the participants mentioned that they do not use any contraceptives at all.

### Factors Associated with Contraceptive Use among Sexually Active WLHIV

After adjusting for the seven significant variables in the logistic regression analysis, only four risk factors had a statistically significant association with contraceptive use among sexually active WLHIV. Regarding religion, while women identifying with Islam showed a reduced likelihood of contraceptive use compared to their Christian counterparts, this difference was not statistically significant (Adjusted Odd Ratio (AOR): 0.461, 95% CI: 0.176–1.213, p=0.117). Educational background played a role, with women without formal education being less inclined to use contraceptives compared to those with primary education (AOR: 0.518, 95% CI: 0.162–1.651, p=0.266). The study results show that women with tertiary or secondary education also showed differences in contraceptive use when compared to those with primary education, though these results were not statistically significant.

In terms of marital status, single women had a higher likelihood of contraceptive use than divorced, widowed, or married women; however, this difference did not attain statistical significance. Separated women, on the other hand, were more likely to use contraceptives than single women, but again, this difference was not statistically significant (AOR: 1.799, 95% CI: 0.184–17.619, p=0.614). Employment emerged as a significant factor: employed women were more inclined to use contraceptives than their unemployed counterparts (AOR: 2.150, 95% CI: 1.279–3.612, p=0.004).

Regarding the type of partner, women with steady partners were less likely to use contraceptives than those married, with the difference nearing statistical significance (AOR: 0.102, 95% CI: 0.022–0.485, p=0.067). Yet, women with casual or no partners showed reduced contraceptive use compared to married women, without a significant difference. Notably, the source of contraceptives was influential: women obtaining contraceptives outside the ART center demonstrated a significantly higher tendency to use them compared to those who did not (AOR: 21.483, 95% CI: 7.279–63.402, p=0.00).

Distance to health facilities also influenced contraceptive use, with an increase in usage as the distance from these facilities grew. Specifically, the distance between 2 and 3 kilometers from the health facilities was significant compared to the reference (AOR: 4.021, 95% CI: 1.343–12.036, p=0.020). Additionally, payment for services was a determinant: women paying for services showed a significantly higher likelihood of using contraceptives (AOR: 14.343, 95% CI: 2.705–76.051, p=0.020). Lastly, concerning adverse reactions to contraceptives, the odds ratio indicated a diminished likelihood of experiencing an adverse reaction (AOR: 0.006, 95% CI: 0.002–0.20), suggesting that women not reporting adverse reactions had significantly lower odds of having them compared to the reference group. (See Table 3)

## DISCUSSION

The study underscored the significant use of contraceptives among sexually active WLHIV in Ibadan, Nigeria, mirroring the contraceptive adoption rates seen in Ethiopia, where 75% of sexually active WLHIV used contraceptives while the remaining 25% faced unmet contraceptive requirements. [17, 22, 23] This utilization of contraceptives plays a fundamental role in advancing family planning goals and reinforcing the Prevention of Mother-To-Child Transmission programs.[24] Yet, the contrasting findings from Oyo State, Nigeria, remind us that awareness does not always lead to action. Even with high contraceptive knowledge levels among WLHIV, usage rates remained disappointingly low [10]; male condoms were most popular, trailed by pills and female condoms.

Conversely, less conventional methods like male and female sterilization and herbal mixtures were minimally favored. Compared to data from Togo, these rates were marginally lower.[25] Although dual contraceptive methods could effectively prevent unwanted pregnancies and sexually transmitted infections (STIs), their adoption was overshadowed by the preference for single methods.[26] Several factors are pivotal in determining contraceptive choices.[27–29] Employment status stood out, corroborated by Banten Province, Indonesia’s findings, which identified employment as a critical influencer.[30] Similarly, the absence of side effects encouraged continuity in contraceptive use, emphasizing the value of a smooth experience.

Contrastingly, the necessity of payments acted as a deterrent, with a study from Uganda linking payment barriers to reduced contraceptive use.[28] Nevertheless, the challenges faced by WLHIV are multi-layered. Sociocultural dynamics heavily influence contraceptive choices.[31] In many African settings, reproductive choices are often determined more by a woman’s partner than by herself. Moreover, the stigma attached to HIV often discourages WLHIV from availing contraceptive services, particularly if healthcare professionals hold biased views.[31–34]

The types of available contraceptives also matter. The dominant use of male condoms, while indicative of their dual protective nature against STIs and pregnancies, also shows a limitation in contraceptive choices. There is an evident need for long-acting methods like IUDs and implants, offering women greater autonomy, but their accessibility is often constrained by availability or cost.[35–37] The role of healthcare infrastructure is undeniable. The inconsistency in contraceptive stock can discourage WLHIV from relying on specific methods, prompting them to settle for less preferred options. Integrating HIV care with contraceptive services might present a more consistent and comprehensive solution.[10]

Comprehensive counseling provides essential information about contraceptive options, side effects, and effectiveness and can significantly guide WLHIV in making informed choices. This approach can demystify misconceptions and align contraceptive choices with individual reproductive and health goals. [38, 39] Lastly, policy frameworks are pivotal. For effective contraceptive adoption among WLHIV, governments, and health bodies must craft policies prioritizing their unique challenges, encompassing contraceptive procurement, training of healthcare professionals, and robust monitoring mechanisms. In essence, while global data paints an overarching picture, addressing the contraceptive needs of WLHIV requires a deeper understanding of the intricate blend of personal, sociocultural, and structural factors. Only a holistic approach, cognizant of these intricacies, can genuinely champion the reproductive rights of every woman.

Self-reported data were used, which may be affected by social desirability and recall bias. The study only focused on women living with HIV and did not include men living with HIV, which limits the understanding of the contraceptive needs of male partners of women living with HIV. The study did not explore the impact of cultural and religious beliefs on contraceptive use, which could significantly influence contraceptive use among this population. The study’s strengths include a large representative sample of the three facilities and participants, including WLHIV, which was possible due to the nature of the facilities that participated in the study.

In evaluating the reproductive health choices of WLHIV, our study traverses a landscape underscored by the United Nations’ Sustainable Development Goals (SDGs).[40] The significance we attribute to contraceptives for enhancing overall health and the crucial PMTCT resonates deeply with the ethos of SDG 3: Good Health and Well-being.[41] Delving into the sociocultural dynamics impacting WLHIV’s reproductive decisions and their multifaceted challenges, our findings mirror SDG 5: Gender Equality [42], which brings to the forefront the pressing narratives of SDG 10, highlighting the stark inequalities faced by WLHIV compared to the broader population.[41, 43] Furthermore, our proposition for integrated and cooperative policy frameworks, tailored by governments and health bodies alike, echoes the collaborative spirit of SDG 17: Partnerships for the Goals.[43, 44] Through the lens of these SDGs, our study amplifies the need for an all-encompassing approach to uphold and advocate for the reproductive rights of WLHIV genuinely

## CONCLUSION

This study provides evidence that shows high levels of contraceptive use among sexually active WLHIV. However, the study identified the need for greater uptake of dual contraceptive methods to reduce the risk of unwanted pregnancy and HIV re-infection among WLHIV. The study also highlighted various factors, such as employment status, access to contraceptive methods, payment for service, and previous experience with contraceptive use, that influence the use of contraceptives among this population. These findings have important implications for policymakers and healthcare providers seeking to improve reproductive health outcomes and reduce the burden of HIV among WL HIV in Nigeria.

## Figures and Tables

**Figure 1:**
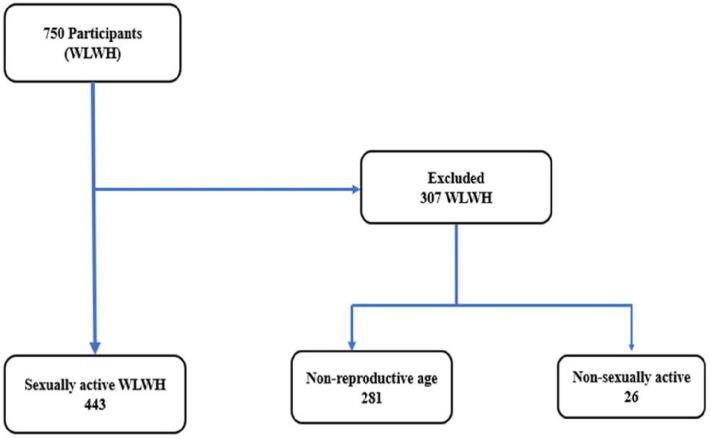
Flow Chart Illustrating the Eligibility Process to Obtain The Final Sample For Analysis of Sexually Active WLHIV

**Figure 2:**
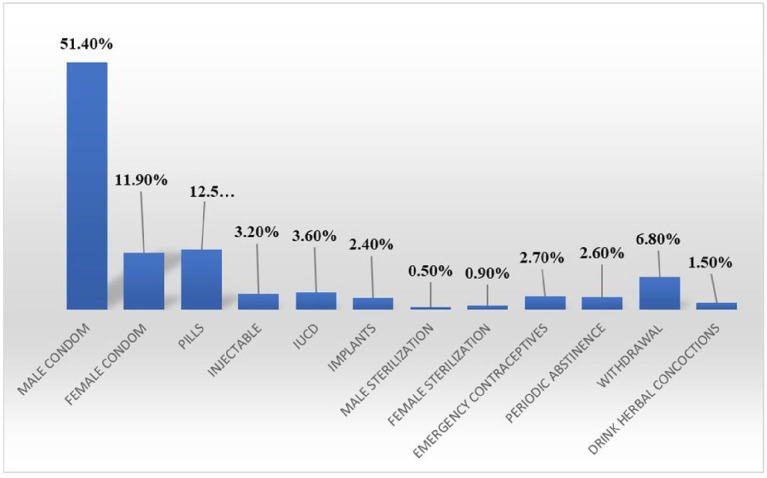
Legend not included with this version.

**Table 1.** Socio-demographic Characteristics of Study Participants (n =433)

	Contraceptive User N=324 [73.1%]	Non-users of Contraceptives N=119 [26.9%]

Variable	N [%]	N [%]

**Age**		

Mean ± SD	36.62±6.70	37.59 ± 7.20

**Marital Status**		

Married	290 [74.6]	99 [25.4]
Divorce	7 [70.0]	3 [30.0]
Widowed	9 [56.2]	7 [43.8]
Separated	8 [66.7]	4 [33.3]
Single	10 [62.5]	6 [37.5]

**Ethnic Group**		

Yoruba	285 [75.0]	95 [25.0]
Igbo	25 [62.5]	15 [37.5]
Hausa	6 [50.0]	6 [50.0]
Others	8 [72.7]	3 [27.3]

**Religion**		

Christianity	188 [72.0]	73 [28.0]
Islam	136 [74.7]	46 [25.3]

**Educational Level**		

Primary level	93 [78.8]	25 [21.2]
Secondary level	135 [67.8]	64 [32.2]
Tertiary level	65 [78.3]	18 [21.7]
None	31 [72.1]	12 [27.9]

**Employment Status**		

Employed	273 [76.5]	84 [23.5]
Unemployed	51 [59.3]	35 [40.7]

**Type of Partner**		

Spouse	269 [74.9]	90 [25.1]
Steady	32 [80.0]	8 [20.0]
Causal	14 [53.8]	12 [46.2]
None	9 [50.0]	9 [50.0]

**Monthly Income**		

< 44.71 USD (NGN 33,000)	225(77.6)	65(22.4)
≥ 44.71 USD (NGN 33,000)	99(64.7)	54(35.3)

**Know Partner’s Status**		

Yes	276 [76.9]	83 [23.1]
No	48 [57.1]	36 [42.9]

**If Yes, Partner Status**		

Positive	111 [77.1]	33 [22.9]
Negative	161 [78.2]	45 [21.8]
Not Applicable	52 [55.9]	41 [44.1]

**Table 2:** Level of Dual Contraceptive Utilization

Variable	N [%]
Dual Contraceptives Usage	136 [30.7]
Single Contraceptive Usage	188 [42.4]
No Usage	119 [26.9]

**Table 3:** Factors Affecting the Use of Contraceptives among Sexually Active Women Living with HIV

Variables	Adjusted Odd Ratio (95% CI)	P value

**Religion**		

Christianity	0.461(0.176, 1.213)	0.117
Islam	Ref	Ref

**Education Level**		

Primary Level	0.518 (0.162, 1.651)	0.266
Secondary Level	1.405(0.438, 4.511)	0.380
Tertiary Level	0.354(0.086,1.448)	0.058
None	Ref	

**Marital status**		

Married	0.661(0.162,1.651)	0.266
Divorce	0.490(0.100,2.410)	0.380
Widowed	0.184(0.032,1.062)	0.585
Separated	1.799(0.184,17.619)	0.614
Single	Ref	

**Employment Status**		

Unemployed	2.150(1.279,3.612)	0.004
Employed	Ref	

**Type of Partner**		

Spouse	0.498(0124,1.993)	0.233
Steady	0.102(0.022,0.485)	0.067
Causal	0.338(0.035,3.300)	0.062
None	Ref	

**Get Contraceptives outside the ART Center**		

Yes	21.483(7.279,63.402)	0.00
No	Ref	

**Distance to the health facilities**		

½ to 1 kilometer	2.669(0.949,0.949)	0.639
2 to 3 kilometres	4.021(1.343,12.036)	0.304
4 to 5 kilometres	Ref	

**Payment for Services**		

Yes	14.343(2.705,76.051)	0.020
No	Ref	

**Adverse Reaction while using Contraceptive**		

Yes	Ref	
No	0.006(0.002,0.20)	0.000

## Data Availability

The data used to support the findings of this study are available from the corresponding author upon request.
